# Clinical Reports

**Published:** 1893-03

**Authors:** E. J. Beall

**Affiliations:** Philadelphia, Pa.; Fort Worth, Texas


### CLINICAL REPORTS.

### LETTER FROM DR. E. J. BEALL.

PHILADELPHIA, PA.

*Editor Daniel's Texas Medical Journal:*

Some one has said that an absent minded person is either a genius or a fool. As I have undertaken to comply with a promise made when in your city, you will not place me in above category.

Dr. Walker and I have, as you know, been at work among the medical men here for some weeks. You are aware that this is and always has been quite a city for doctors. In some streets, notably Walnut, every second or third house bears a "wee sign," indicating that human ills are looked after within. I have been very much struck with the diminutiveness of physicians' signs in this city, and have wondered whether or not Philadelphia doctors were not readers of the *London Lancet*, because several years ago the editor of that journal wrote, "the larger the sign the smaller the doctor," and as many of the doctors from Shippen to the present time have been considered "big" doctors, ergo the "wee" sign.

I have not straightened up my notes yet, hence I will not undertake to write you anything of an especially practical nature this time; will write briefly, only that you cannot say I'm a "genius or a fool," reserving for a subsequent letter men and motives as I see them in my daily rounds in the Quaker city, and more especially something of that wonderful man, Dr. Jos. Price and his work, with whose services are connected, and who McGuin, McMertry and others claim stands to-day without a peer on earth in his special line of work.

As you know, Philadelphia was for more than a century considered the medical center of America. You and I can recall medical men, now passed away, who rode horseback to Philadelphia from the old States, to seek knowledge at her time-honored medical altars, in the old days when the civilizing influences of railroads were limited, when minds were fired with new thoughts by the slow process of the mail coach, when new ideas could not be whispered from New York to Chicago in an instant. It is well known that in recent years Philadelphia has lost some of the prestige she so long enjoyed, that a rival city craved that prestige, worked for it and has measurably succeeded. Philadelphia has realized that another city has attracted professional men and students to an extent that creates alarm at the waning fame of the old medical center of Quaker quiet and morals. There is no question but that Philadelphia has lost caste as a medical center. The fame established by Rush, Homer, Jackson, Chapman, Dewes, Eberle; later, Wood, Gross, Smith and others, as such characters burnished the decades amid which they lived and acted, is being dimmed by fires that burned upon the altars. Should another city wrest the name of medical center from Philadelphia, her glory will yet live in the history of the University and her more than century of life. The Jefferson, with her two-thirds century age, will live in memories, museums and monuments. The impress of her love and greatness will live; if dimmed, there will still linger the halo of former prestige and precedent, to gild her history for all time. Another city may seize the torch illuminating work and deed and press forward, but the spark that created the blaze, the germ that ripened into the colony, will not be forgotten in the the race of human progress. Why should Philadelphia, after an uninterrupted march of medical supremacy and greatness, allow her laurels to be dimmed or wrested from her? Her capitalists, her philanthropists, the great State of Pennsylvania had coached her hospitals and her schools. Gerard, Drexel and others had aided by pre mortem monuments, preliminary education. Money had been lavishly poured by her humanitarians into the coffers of her schools and her hospitals. This question, one of Philadelphia's rising medical men shall answer. I will not venture to account for the decline of Philadelphia, if indeed a decline exists. The memory of my lamented father, an allumuus of the University of Pennsylvania of the class of '31, would interdict a personal expression. The decline of empires of national greatness has been the theme of the historian from Adam till the present time. The political and religious phases of past centuries has been studied as to the influences that have demoralized and stayed the advance of science, art and literature. I cannot review these. I am interested briefly why, if such is the case, has Philadelphia waned as a medical center? and without a personal conclusion, present the subject to the student who would study the cause of the rise and fall of literature as applied to Philadelphia or other city or nation, as well as the study of the means needed to prevent such decline.

Reputation has been likened to a bubble. Bubbles from molecular changes will burst. The reputation or the foundation laid by the makers, it is said, did not withstand certain changes wrought by time, as was told me. That although Philadelphia capitalists had espoused cupidity with progress, though bountiful wills, old Blockley, the Presbyterian, the German, the Jefferson and other hospitals, had their coffers filled; alas, the chivalry of the profession lacked its pristine strength as compared with the assistance given by the *populus*. The profession made a trade of their calling; their Philadelphia medical greatness waned. You can picture the rocks that would arise under such circumstances to cause the wreck of her former glory. You are aware that there are great medical minds and teachers in Philadelphia. Large classes are found in her old schools—800 at the University of Pennsylvania—yet the curriculum has been advanced quite recently to four years. We may think of this matter in another light; that this city maintains her ancient attractions for students, but other cities have come to the front as candidates for teachers and have assumed enviable positions, not because of Philadelphia's decadence, but because of an increase in the material out of which doctors are made. Yet one cannot but be impressed with the idea that a firm belief exists that Philadelphia is not maintaining the proud position she once occupied as a teachers' center. This may, however, not exist in fact, but may grow out of the essence in the character of the green-eyed monster.

There is an excellent post graduate school here—not so well attended, perhaps, as the same character of schools in New York, but the facilities are good and the teachers as able and zealous as any in the land.

There is an association of physicians and surgeons recently organized, intended to show courtesies and attentions to visiting physicians to the Quaker city. A card furnished by Dr. Wharton, the secretary, will introduce the holder to the ablest men here, and to their work in the various hospitals of the city. I have seen some very fine work by Deaver, the Mortons, Baldy and others. I attended with great pleasure the clinics of the affable and learned Weir Mitchell and listened with much pleasure to Pepper, DeCosta and others.

FORT WORTH, TEXAS, February, 1893.

P. S. Since my return home from New York, whither Walker and I went after our stay in Philadelphia, I have been so overwhelmed with work incident to the prevailing influenza, its complications and sequences, I have not had time to prepare a communication based upon the material gleaned during my absence. This I promise in the near future.

Yours, etc.,

E. J. BEALL.

